# Toward the Decarbonization of Plastic: Monopolymer Blend of Virgin and Recycled Bio-Based, Biodegradable Polymer

**DOI:** 10.3390/polym14245362

**Published:** 2022-12-08

**Authors:** Vincenzo Titone, Maria Chiara Mistretta, Luigi Botta, Francesco Paolo La Mantia

**Affiliations:** 1 Department of Engineering, University of Palermo, Viale delle Scienze, 90128 Palermo, Italy; 2 INSTM Consortium for Materials Science and Technology, Via Giusti 9, 50125 Firenze, Italy

**Keywords:** biodegradable polymers, mechanical recycling, monopolymer blends, decarbonization

## Abstract

Decarbonization of plastics is based on two main pillars: bio-based polymers and recycling. Mechanical recycling of biodegradable polymers could improve the social, economic and environmental impact of the use of these materials. In this regard, the aim of this study was to investigate whether concentrations of the same recycled biopolymer could significantly affect the rheological and mechanical properties of biodegradable monopolymer blends. Monopolymer blends are blends made of the same polymers, virgin and recycled. A sample of commercially available biodegradable blend was reprocessed in a single-screw extruder until two extrusion cycles were completed. These samples were exposed to grinding and melt reprocessed with 75% and 90% of the same virgin polymer. The blends were characterized by tensile tests and rheological tests. The results obtained showed that while multiple extrusions affected the mechanical and rheological properties of the polymer, the concentration of the reprocessed material present in the blends only very slightly affected the properties of the virgin material. In addition, the experimentally observed trends were accurately predicted by the additive model adopted.

## 1. Introduction

In recent years, the large increase in world population, combined with the need to improve lifestyles, has led to a dramatic increase in polymer consumption [1,2,3].

Fossil-derived polymers make a significant contribution to the anthropogenic carbon dioxide emission released to the environment. This contribution derives from all the steps of the life of the polymer: extraction of the oil, cracking, polymerization, processing and end of life [4,5]. The decarbonization of plastics can be achieved through two important steps: use of bio-based, or at least partly bio-based, polymers and recycling of these bio-based polymers. The end of life of these polymers, both through biodegradation or through combustion to recover energy, does not put new carbon dioxide in the environment and, rather, carbon dioxide is used for the production of the biomasses to be used for bio-based polymers. Moreover, the mechanical recycling [6,7], extending the life of the polymers, can, in its turn, make a significant contribution to a decrease in the huge amounts of plastics.

To date, although the recycling of conventional polymers is quite well researched [8,9,10,11], there is less research on the recycling of biodegradable polymers.

In our previous work [12], the rheology of biodegradable poly(butylene adipate-co-butylene terephthalate) (PBAT) blends subjected to multiple extrusion cycles, as well as the mechanical properties, was studied. The results showed that the conditions used had no significant effect on the rheological and mechanical properties of the sample; therefore, the material was considered reusable. Similar studies on poly(lactic acid) (PLA) [13,14,15] showed that it is possible to obtain recycled PLA with good properties only by adding small amounts of additives during mechanical recycling. The study by Bruzaud et al. [16] showed that PLA is more stable than PHBV and, also, when blended with PHBV, shows more stability than pure PHBV in terms of recyclability. La Mantia et al. [17] showed that typical processing conditions begin to significantly affect the rheological and mechanical properties of starch-based biodegradable polymers only after five extrusions.

Studies on monopolymer blends [18,19,20,21] reported that the mechanical and rheological properties of these blends were in most cases intermediate between those of the two components used alone.

Bio-Flex^®^ is a commercial blend of poly(lactic acid) PLA and poly(butylene adipate-co-terephthalate) (PBAT) [22] used in various applications. This polymer is biodegradable although not fully bio-based. Due to its physical properties, it is generally considered the biodegradable alternative to low-density polyethylene; moreover, it is one of the new PLA-based materials approved for food contact [23] and thus commercially available with wide use in food packaging.

In this paper, a sample of commercially available Bio-Flex^®^ F2110 was reprocessed up to two times using a single-screw extruder. The main purpose was to determine the effect of small amounts of reprocessed polymer on the rheological (shear and elongation) then mechanical properties of monopolymer blends. In addition, experimental values of mechanical properties were compared with theoretical values obtained using an additive model proposed in our work [24].

The results obtained demonstrate that while multiple extrusions significantly affect the mechanical and rheological properties, the concentration of reprocessed material present in the blends very slightly affect the virgin material. These results indicate that the properties of the monopolymer blends were similar to the virgin material. In addition, the experimentally observed trends were accurately predicted by the additive model.

## 2. Materials and Methods

### 2.1. Materials

The material used in this work was a Bio-Flex^®^ F2110 supplied from FKuR Kunststoff GmbH (Willich, Germany) with the principal characteristics given in Table 1 [25].

### 2.2. Processing

After drying in a vacuum oven for 24 h [26], commercial Bio-Flex^®^ F2110 (BF) and reprocessed (BF_RE1_ and BF_RE2_) pellets, mixed with the weight compositions listed in Table 2, were extruded with a single screw extruder (Thermo Scientific HAAKE PolyLab QC, Karlsruhe, Germany). The temperature profile was set to 150–160–170–180 °C, while the rotational speed of the screw was 60 rpm.

The first number indicates the number of processes, the second number the percentage of recycled material. The resulting blends had the following compositions, see Table 3.

The compositions of the blends were obtained from the following equations:

(1) R_1_ = xBF + (1 − x)BF_RE1_

(2) R_2_ = xBF + (1 − x)[xBF_RE1_ + (1 − x)BF_RE2_]

where x is the weight fraction of the assumed polymer.

**Figure 1:**
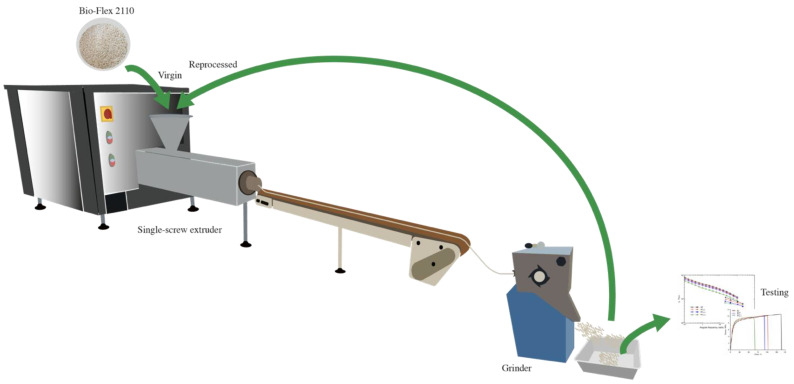
Illustrative image of the procedure used in this work.

Figure 1 illustrates the process used in this work.

Rheological and mechanical characterization was performed on compression molded samples obtained using a Carver laboratory hydraulic press (Carver, Wabash, IN, USA) at a temperature of 180 °C with a mold pressure of 300 psi, for about 3 min. Before compression molding, the pellets were dried in a vacuum oven under the same conditions as above.

### 2.3. Characterization

#### 2.3.1. Rheological Characterization

An ARES G2 rotational rheometer (TA Instruments, New Castle, DE, USA) was used to perform rheological characterization in shear flow. The experiments were carried out in parallel plates with a gap of about 1.5 mm and a diameter of 25 mm. The shear viscosity values of the samples were measured from 100 to 0.1 rad/s at 180 °C.

A capillary viscometer (Rheologic 1000, CEAST, Turin, Italy) operating at 180 °C was used for capillary rheological tests to obtain flow curves in agreement with the power law equation.

The capillary used in these tests had a length-to-diameter ratio (L/D) of 40. Thus, due to the high length-to-diameter ratio, Bagley’s correction was not applied, while Rabinowitsch’s correction was applied.

As for elongational viscosity, to correlate this behavior to the typical processing operations, non-isothermal elongational flow tests were performed in the same instrument equipped with a tensile module. The force at break, known as melt strength (MS), of the melt filament was read directly, while the BSR, i.e., the ratio of the drawing speed at break to the extrusion speed at the die, was calculated as follows (Equation (3)):

(3) $$\mathrm{BSR}=\frac{V_{\mathrm{roll}}}{V_{p\;}\cdot \;\frac{D_{p}^{2}}{D_{c}^{2}}}$$

where V_roll_ is the collecting speed, V_p_ is the capillary piston speed, D_p_ is the piston diameter, and D_c_ is the diameter of the capillary.

#### 2.3.2. Mechanical Characterization

Tensile tests were carried out using an Instron Universal Testing Machine (Instron, Norwood, MA, USA) mod. 3365 equipped with a 1 kN load cell, and operating at a crosshead speed of 1 mm/min for up to 3 min; then, the crosshead speed was increased to 100 mm/min until specimen failure.

Elastic modulus (E) was calculated as the initial slope of the stress–strain curves, while tensile strength (TS) and elongation at break (EB) were evaluated as maximum stress and strain values for each curve, respectively.

#### 2.3.3. Cross-Linking Investigation

The presence of cross-linking was assessed by measuring the residual gel fraction (GF), obtained after extraction in chloroform for 24 h.

### 2.4. Statistical Analysis

Statistical analyses of the data were performed by one-way analysis of variance. Student’s *t*-test was used to calculate differences between the means, with a significance level of *p* < 0.05.

## 3. Results and Discussion

### 3.1. Characterization of Virgin and Recycled BF

**Figure 2:**
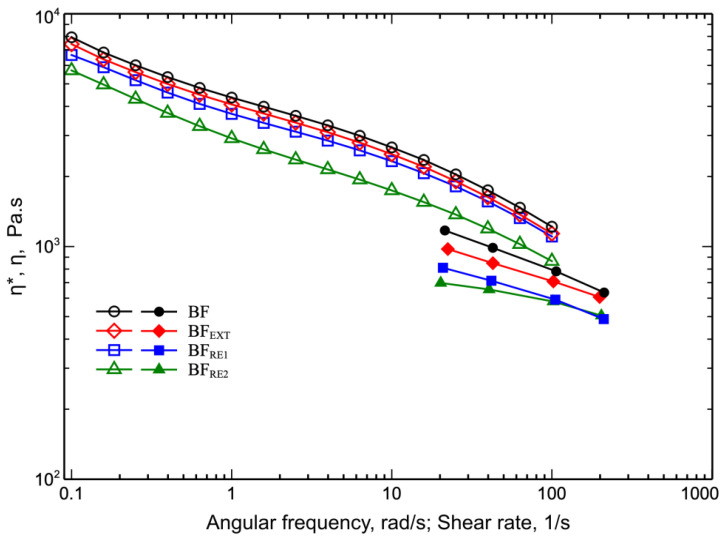
Flow curves of virgin and extruded BF sample, and samples reprocessed 1 and 2 times. Data taken from rotational rheometer (open symbols) and capillary viscometer (closed symbols).

In Figure 2, the flow curves measured from rheological tests in a rotational rheometer (complex viscosity, η* vs. angular frequency) and in a capillary viscometer (shear viscosity, η vs. shear rate) for virgin and samples reprocessed one and two times are shown.

As shown in Figure 2, as the reprocessing steps increased, the viscosity of the reprocessed samples decreased over the entire investigated frequency or shear rate. The results showed that the molecular weight of BF samples decreases during reprocessing because of the thermomechanical stress acting on the melt. In addition, it can be observed that the viscosity curves measured by the rotational rheometer and the flow curves measured by the capillary viscometer do not superimpose. This means that the Cox–Merz rule is not obeyed. This result has been found in other polymer systems and is explained by considering the heterogeneous nature of these polymeric systems [13,27,28]. Finally, the flow curves do not reach a Newtonian plateau and, rather, an upturn is observed at the lower frequencies. This seems to suggest that some filler is present in the polymer system [29].

**Figure 3:**
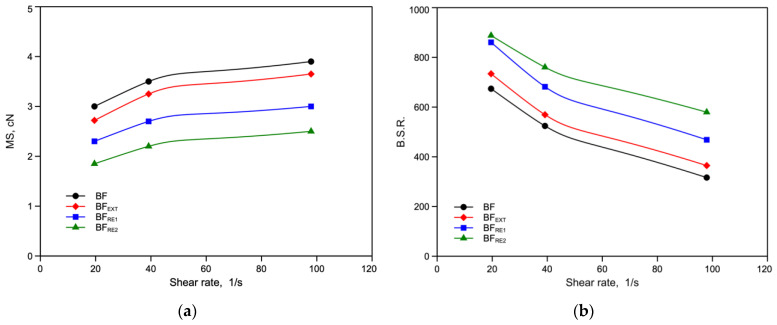
(**a**) Melt strength (MS) and (**b**) breaking stretching ratio (BSR) as functions of the shear rate of virgin and extruded BF sample and samples reprocessed 1 and 2 times.

The rheological behavior in non-isothermal elongational flow of the reprocessed samples was monitored, and the measured melt strength (MS) and stretching to break ratio (BSR) are shown in Figure 3a,b.

The melt strength decreases with increasing reprocessing steps. These results are in complete agreement with the shear viscosity results. The BSR curves present a mirror image of the MS curves in that they decrease with increasing shear rate. The BSR values increase with the number of extrusions. This is due to the decrease of the molecular weight that gives rise to a more deformable melt.

**Figure 4:**
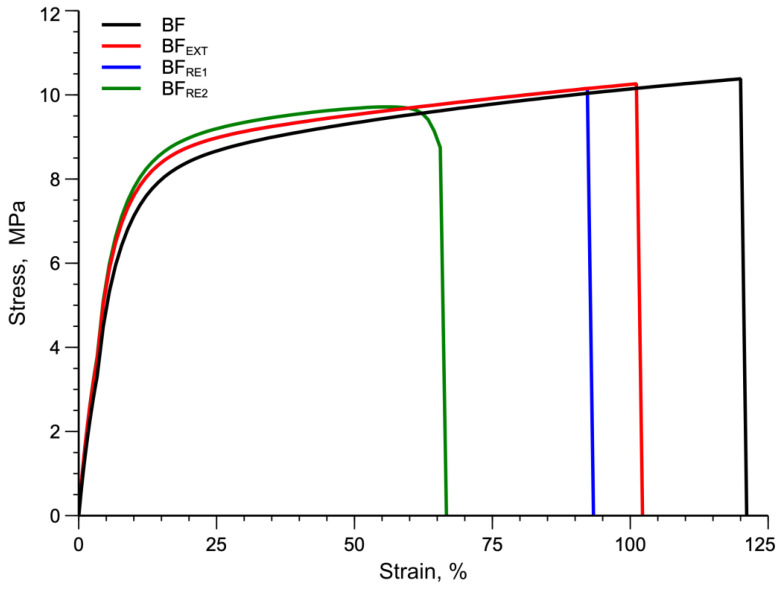
Comparison of stress–strain curves for virgin and extruded BF sample and samples reprocessed 1 and 2 times.

The stress–strain curves for virgin material and samples reprocessed one and two times are shown in Figure 4, and the tensile properties resulting from the tests are summarized in Table 4.

It can be seen that the number of extrusion cycles has a clear influence mainly on the elongation at break, while less relevance is observed in the elastic modulus and in the tensile strength. In more detail, the elongation at break of sample BF was 121 ± 21%, while BF_RE1_ and BF_RE2_ show elongations at break values of 93.9 ± 24% and 72.8 ± 18%, respectively. The tensile strength, for all samples, remained almost the same. On the contrary, the elastic modulus of the virgin sample 123 ± 10 MPa of BF increased to 140 ± 12 MPa and 144 ± 10 MPa for BF_RE1_ and BF_RE2_, respectively (see Table 4).

This result is expected because, based on the results obtained from the flow curves, a decrease in molecular weight leads to an increase in crystallinity [24] and thus an increase in elastic modulus.

### 3.2. Characterization of the Monopolymer Blends

**Figure 5:**
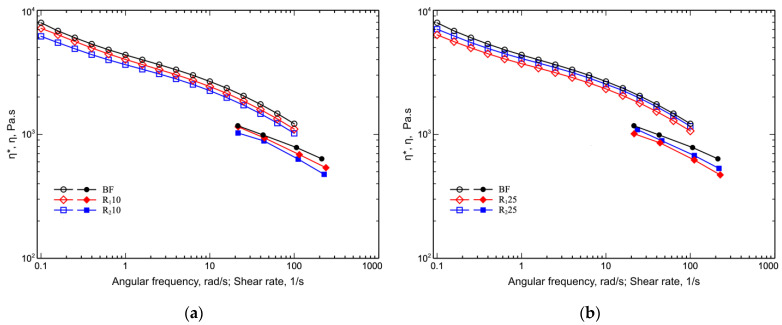
Flow curve of virgin BF sample and reprocessed blends: (**a**) BF, R_1_10 and R_2_10; (**b**) BF, R_1_25 and R_2_25.

In Figure 5a,b the flow curves of virgin BF sample and reprocessed blends (see Table 2) are reported.

A decrease in viscosity was observed as the amount of reprocessed material for the R_1_10 blends increased (see Figure 5a and Table 5).

This behavior is, of course, due to a reduction in molecular weight and thus a reduction in the viscosity of the reprocessing components in the blend. In the 25% blends the behavior is slightly different; in fact, in this case the reduction in the R_2_25 blend is less than in the R_1_25% blend. This change can be attributed to the formation of branched structures. In fact, BF, as already reported [30], tends to form cross-links during processing; however, since no cross-linked structures were found and since branching is the first step in the formation of cross-linked structures, the increase in viscosity of the R_2_25 blend compared to the R_1_25 blend can only be attributed to the presence of branched macromolecules formed due to thermomechanical stresses.

**Figure 6:**
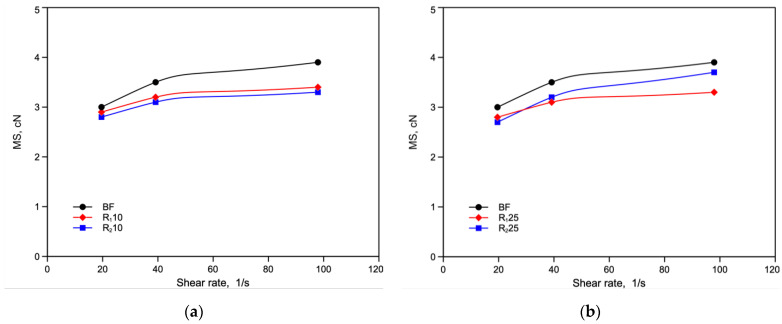
Melt strength (MS) as function of the shear rate of virgin BF sample and reprocessed blends: (**a**) BF, R_1_10 and R_2_10; (**b**) BF, R_1_25 and R_2_25.

**Figure 7:**
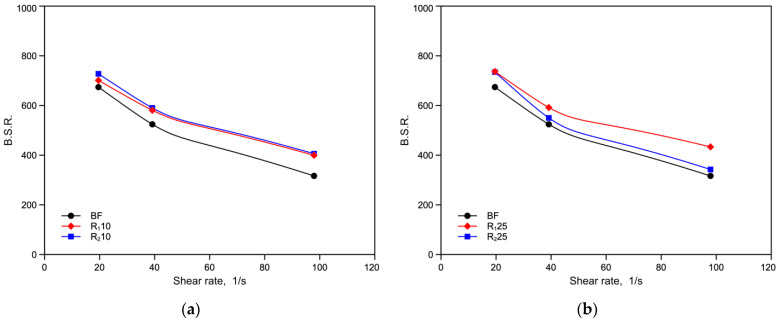
Breaking stretching ratio (BSR) as function of the shear rate of virgin BF sample and reprocessed blends: (**a**) BF, R_1_10 and R_2_10; (**b**) BF, R_1_25 and R_2_25.

Figure 6a,b and Figure 7a,b show the values of melt strength (MS) and breaking stretching ratio (BSR), respectively, for all the monopolymer blends as a function of shear rate.

The MS values of the blends are lower than those of the virgin polymer due, as already reported [12], to the decrease in molecular weight of the recycled component. However, it can be seen that the R_2_25 blend shows higher melt strength values than R_1_25, while the opposite is true for BSR. This effect confirms the previous hypothesis of formation of branching, because the branching increases the MS and decrease BSR. Although this increase of viscosity has not been observed for the BF_RE2_ sample, it is worth mentioning that in the monopolymer blend the polymer undergoes a further extrusion and then further degradation that can give rise to formation of branching. No test is possible to measure this branching due to the very low amount of the twice recycled component.

**Figure 8:**
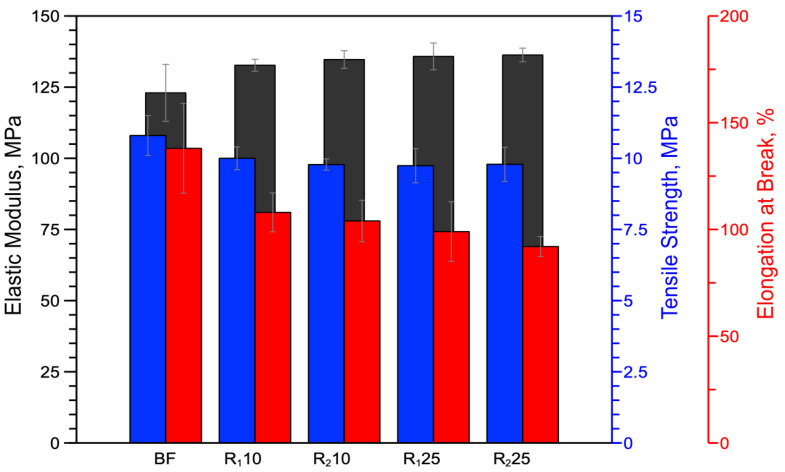
Bar chart of elastic modulus, tensile strength and deformation at break of virgin BF sample and reprocessed blend.

Figure 8 and Table 6 show the results of mechanical tests performed on the monopolymer blends.

The bar chart shows that, as the amount of reprocessed material increases, there is a slight increase in elastic modulus. In more detail, the maximum increase in elastic modulus compared to virgin polymer is about 10%. Tensile strength remains almost unchanged for all blends (see Table 6). In contrast, elongation at break shows a maximum decrease of about 24%.

### 3.3. Additive Model

As explained previously in our paper [24], if no drastic change in chemical nature and morphology occurs during reprocessing, the two components will be miscible, thus the properties can be estimated as the weighted sum of the properties of the individual components present in the monopolymer blends. However, if properties such as tensile strength can be measured only after some kind of processing for the production of the specimens, the properties of the virgin polymer cannot be determined and the preparation of the specimens involves a melt process. Therefore, in this case, it was assumed that:

(4) BF ≃ BF_EXT_

Then, a specific property, P, of the monopolymer blend can be evaluated as:

(5) P(R_1_) = xBF_EXT_ + (1 − x)BF_RE1_

(6) P(R_2_) = xBF_EXT_ + (1 − x)[xBF_RE1_ + (1 − x)BF_RE2_]

where x is the weight fraction of the assumed virgin polymer.

**Figure 9:**
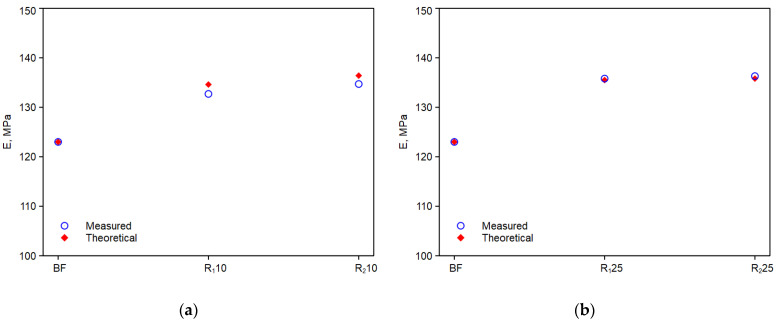
Experimental and theoretical curves obtained from equation above of the elastic modulus: (**a**) R10; (**b**) R25.

**Figure 10:**
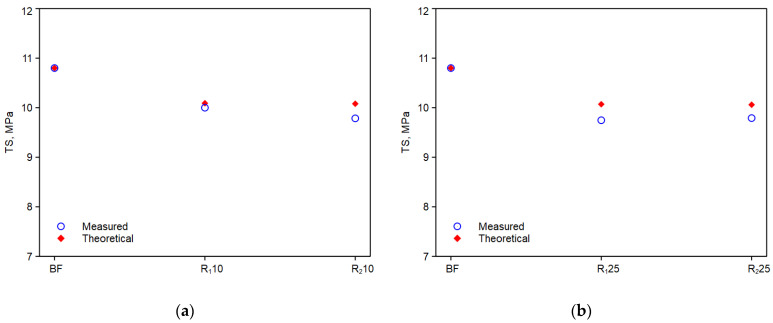
Experimental and theoretical curves obtained from equation above of the tensile strength: (**a**) R10; (**b**) R25.

**Figure 11:**
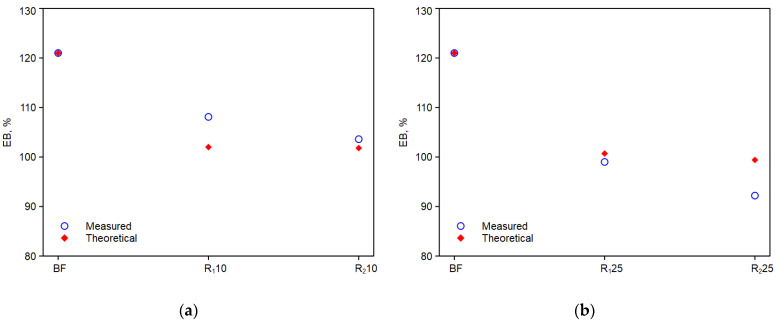
Experimental and theoretical curves obtained from equation above of the elongation at break: (**a**) R10; (**b**) R25.

Figure 9a,b, Figure 10a,b and Figure 11a,b show the trends of the elastic modulus, E, tensile strength, TS, and elongation at break, EB, of the experimental values with the theoretical values calculated with the above equations.

As it can be observed, the comparison with experimental values is good. Thus, the adopted model can predict the mechanical property values of these monopolymer blends quite effectively, provided that the chemical nature and morphology of the reprocessed components do not change significantly [31].

## 4. Conclusions

In this study, a commercial biopolymer called Bio-Flex^®^ F2110 was reprocessed in a single extruder up to two times. Then, a fraction was ground and reprocessed, at 10 and 25 wt%, with the same virgin polymer, simulating a classic industrial recycle operation. The mechanical and rheological properties in shear and elongation flow were evaluated. The results obtained showed the predominance of the chain scission mechanism, with the exception of the R_2_25 blend, which appeared to show both chain scission behavior and branching. The mechanical results showed the great recyclability of this polymer system in monopolymer blends, as the rheological and mechanical property values remain almost constant compared to the virgin material. Finally, the additive model adopted predicts the behavior of these monopolymer blends quite well.

## Figures and Tables

**Table 1:** Principal properties of Bio-Flex^®^ F2110 used in this study.

Name	Density, g/cm^3^	Melting Temperature, °C	MFI, g/10 min	Weight-Average Molecular Weight, kD
Bio-Flex^®^ F2110	1.27	153	6 ^a^	53

^a^ 190 °C and 2.16 kg.

**Table 2:** Composition of the investigated blends.

Blend Name	BF, %	BF_RE1_, %	R_1_, %
R_1_10	90	10	-
R_2_10	90	-	10 ^a^
R_1_25	75	25	-
R_2_25	75	-	25 ^b^

^a^ R_1_10, ^b^ R_1_25.

**Table 3:** Composition of the investigated blends.

Blend Name	BF, %	BF_RE1_, %	BF_RE2_, %
R_1_10	90	10	-
R_2_10	90	9	1
R_1_25	75	25	-
R_2_25	75	18.75	6.25

**Table 4:** Elastic modulus (E), tensile strength (TS) and elongation at break (EB) for virgin and extruded BF sample and samples reprocessed 1 and 2 times.

Name	E, MPa	TS, MPa	EB, %
BF	123 ± 10 ^a^	10.8 ± 0.7 ^a^	121 ± 21 ^a^
BF_EXT_	134 ± 8.3 ^b^	10.1 ± 1.0 ^b^	103 ± 36 ^b^
BF_RE1_	140 ± 12 ^c^	10.0 ± 0.9 ^b^	93.9 ± 24 ^c^
BF_RE2_	144 ± 10 ^c^	9.8 ± 1.2 ^b^	72.8 ± 18 ^d^

Different letters in the same column indicate significant differences (*p* < 0.05) when analyzed by multiple Student’s *t*-tests.

**Table 5:** Viscosity values at 0.1 rad/s (Pa.s) of virgin BF sample and reprocessed blends.

	BF	R_1_10	R_2_10	R_1_25	R_2_25
η* at 0.1 rad/s, Pa.s	7927	7153	6169	6534	6836

**Table 6:** Elastic modulus (E), tensile strength (TS) and elongation at break (EB) of virgin BF sample and reprocessed blend.

Name	E, MPa	TS, MPa	EB, %
BF	123.0 ± 10 ^a^	10.8 ± 0.7 ^a^	121 ± 21 ^a^
R_1_10	132.7 ± 2.1 ^b^	10.0 ± 0.4 ^b^	108 ± 9.1 ^b^
R_2_10	134.7 ± 3.1 ^c^	9.78 ± 0.2 ^c^	104 ± 9.7 ^c^
R_1_25	135.8 ± 4.7 ^d^	9.74 ± 0.6 ^d^	99 ± 14 ^d^
R_2_25	136.3 ± 2.4 ^c^	9.79 ± 0.6 ^d^	92 ± 4.7 ^e^

Different letters in the same column indicate significant differences (*p* < 0.05) when analyzed by multiple Student’s *t*-tests.

## Data Availability

The date presented in this work are available on request from the corresponding author.

## References

[1] Plastics Europe Plastics—The Facts 2019 an Analysis of European Plastics Production, Demand and Waste Data (2019). https://www.plasticseurope.org/it/resources/publications/1804-plastics-facts-2019.

[2] Charles D., Kimman L., Saran N. (2021). The Plastic Waste Maker Index. Revealing the Source of the Single-Use Plastics Crisis Minderoo Foundation. https://www.minderoo.org/plastic-waste-makers-index/.

[3] Sudesh K., Iwata T. (2008). Sustainability of Biobased and Biodegradable Plastics. CLEAN Soil Air Water.

[4] Vollmer I., Jenks M.J.F., Roelands M.C.P., White R.J., Harmelen T., Wild P., Laan G.P., Meirer F., Keurentjes J.T.F., Weckhuysen B.M. (2020). Beyond Mechanical Recycling: Giving New Life to Plastic Waste. Angew. Chem. Int. Ed..

[5] Helmes R.J.K., Goglio P., Salomoni S., van Es D.S., Vural Gursel I., Aramyan L. (2022). Environmental Impacts of End-of-Life Options of Biobased and Fossil-Based Polyethylene Terephthalate and High-Density Polyethylene Packaging. Sustainability.

[6] Hamad K., Kaseem M., Deri F. (2013). Recycling of Waste from Polymer Materials: An Overview of the Recent Works. Polym. Degrad. Stab..

[7] La Mantia F.P. (2004). Polymer Mechanical Recycling: Downcycling or Upcycling?. Prog. Rubber Plast. Recycl. Technol..

[8] Andrews G.D., Subramanian P.M., American Chemical Society (1992). Emerging Technologies in Plastics Recycling.

[9] La Mantia F. (2002). Handbook of Plastics Recycling.

[10] Scheirs J. (1998). Polymer Recycling: Science, Technology, and Applications.

[11] Brandrup J. (1996). Recycling and Recovery of Plastics.

[12] La Mantia F.P., Botta L., Mistretta M.C., Di Fiore A., Titone V. (2020). Recycling of a Biodegradable Polymer Blend. Polymers.

[13] Beltrán F.R., Gaspar G., Dadras Chomachayi M., Jalali-Arani A., Lozano-Pérez A.A., Cenis J.L., de la Orden M.U., Pérez E., Martínez Urreaga J.M. (2021). Influence of Addition of Organic Fillers on the Properties of Mechanically Recycled PLA. Environ. Sci. Pollut. Res..

[14] Beltrán F.R., Infante C., de la Orden M.U., Martínez Urreaga J. (2019). Mechanical Recycling of Poly(Lactic Acid): Evaluation of a Chain Extender and a Peroxide as Additives for Upgrading the Recycled Plastic. J. Clean. Prod..

[15] Barletta M., Aversa C., Puopolo M. (2020). Recycling of PLA-based Bioplastics: The Role of Chain-extenders in Twin-screw Extrusion Compounding and Cast Extrusion of Sheets. J. Appl. Polym. Sci..

[16] Zembouai I., Bruzaud S., Kaci M., Benhamida A., Corre Y.-M., Grohens Y. (2014). Mechanical Recycling of Poly(3-Hydroxybutyrate-Co-3-Hydroxyvalerate)/Polylactide Based Blends. J. Polym. Environ..

[17] La Mantia F.P., Scaffaro R., Bastioli C. (2002). Recycling of a Starch-Based Biodegradable Polymer. Macromol. Symp..

[18] Marrone M., La Mantia F.P. (1996). Monopolymers Blends of Virgin and Recycled Polypropylene. Polym. Recycl..

[19] Wenguang M., La Mantia F.P. (1996). Processing and Mechanical Properties of Recycled PVC and of Homopolymer Blends with Virgin PVC. J. Appl. Polym. Sci..

[20] Valenza A., La Mantia F.P. (1988). Recycling of Polymer Waste: Part II—Stress Degraded Polypropylene. Polym. Degrad. Stab..

[21] Wenguang M., La Mantia F.P. (1995). Recycling of Post-Consumer Polyethylene Greenhouse Films: Monopolymer Blends of Recycled and Virgin Polyethylene. Polym. Netw. Blends.

[22] Gregorova A., Riedl E., Sedlarik V., Stelzer F. (2012). Effect of 4,4′-Methylenediphenyl Diisocyanate on Thermal and Mechanical Properties of Bioflex/Lactic Acid Polycondensate Blends: Effect of midi on Bioflex blends. Asia-Pac. J. Chem. Eng..

[23] Peters S. (2011). Material Revolution. Sustainable and Multi-Purpose Materials for Design and Architecture.

[24] La Mantia F.P., Mistretta M.C., Titone V. (2021). An Additive Model to Predict the Rheological and Mechanical Properties of Polypropylene Blends Made by Virgin and Reprocessed Components. Recycling.

[25] Morreale M., Liga A., Mistretta M., Ascione L., Mantia F. (2015). Mechanical, Thermomechanical and Reprocessing Behavior of Green Composites from Biodegradable Polymer and Wood Flour. Materials.

[26] Titone V., Correnti A., La Mantia F.P. (2021). Effect of Moisture Content on the Processing and Mechanical Properties of a Biodegradable Polyester. Polymers.

[27] Krishnamoorti R., Yurekli K. (2001). Rheology of Polymer Layered Silicate Nanocomposites. Curr. Opin. Colloid Interface Sci..

[28] Ren J., Krishnamoorti R. (2003). Nonlinear Viscoelastic Properties of Layered-Silicate-Based Intercalated Nanocomposites. Macromolecules.

[29] Herschel W.H., Bulkley R. (1926). Konsistenzmessungen von Gummi-Benzollösungen. Kolloid-Z.

[30] Shojaeiarani J., Bajwa D.S., Rehovsky C., Bajwa S.G., Vahidi G. (2019). Deterioration in the Physico-Mechanical and Thermal Properties of Biopolymers Due to Reprocessing. Polymers.

[31] La Mantia F.P., Mistretta M.C., Titone V. (2021). Rheological, Mechanical and Morphological Characterization of Monopolymer Blends Made by Virgin and Photo-Oxidized Polypropylene. Recycling.

